# Evolutionary relationships of *Fusobacterium nucleatum *based on phylogenetic analysis and comparative genomics

**DOI:** 10.1186/1471-2148-4-50

**Published:** 2004-11-26

**Authors:** Alex Mira, Ravindra Pushker, Boris A Legault, David Moreira, Francisco Rodríguez-Valera

**Affiliations:** 1 Evolutionary Genomics Group, División de Microbiología, Universidad Miguel Hernández, Apartado 18, San Juan 03550, Alicante, Spain; 2 UMR CNRS 8079, Ecologie, Systématique et Evolution, Université Paris-Sud, bâtiment 360, 91405 Orsay Cedex, France

## Abstract

**Background:**

The phylogenetic position and evolutionary relationships of Fusobacteria remain uncertain. Especially intriguing is their relatedness to low G+C Gram positive bacteria (Firmicutes) by ribosomal molecular phylogenies, but their possession of a typical gram negative outer membrane. Taking advantage of the recent completion of the *Fusobacterium nucleatum *genome sequence we have examined the evolutionary relationships of *Fusobacterium *genes by phylogenetic analysis and comparative genomics tools.

**Results:**

The data indicate that *Fusobacterium *has a core genome of a very different nature to other bacterial lineages, and branches out at the base of Firmicutes. However, depending on the method used, 35–56% of *Fusobacterium *genes appear to have a xenologous origin from bacteroidetes, proteobacteria, spirochaetes and the Firmicutes themselves. A high number of hypothetical ORFs with unusual codon usage and short lengths were found and hypothesized to be remnants of transferred genes that were discarded. Some proteins and operons are also hypothesized to be of mixed ancestry. A large portion of the Gram-negative cell wall-related genes seems to have been transferred from proteobacteria.

**Conclusions:**

Many instances of similarity to other inhabitants of the dental plaque that have been sequenced were found. This suggests that the close physical contact found in this environment might facilitate horizontal gene transfer, supporting the idea of niche-specific gene pools. We hypothesize that at a point in time, probably associated to the rise of mammals, a strong selective pressure might have existed for a cell with a Clostridia-like metabolic apparatus but with the adhesive and immune camouflage features of Proteobacteria.

## Background

The genus *Fusobacterium*, together with some close relatives such as *Leptotrichia*, forms an ecologically and physiologically coherent group [1]. They seem to be inhabitants of the mammal gastrointestinal tract probably specialized in the oral cavity. Specifically, they are components of the dental plaque, a highly complex habitat that has received considerable attention in recent years due to its involvement in dental pathology [2]. They are all fermentative anaerobes that use mostly peptides as their energy source (see, for example, [3]). The species *Fusobacterium nucleatum *has received particular attention being a key component of the human dental plaque that also has considerable pathogenic potential. In fact after *Bacteroides*, *Fusobacterium *is responsible for most human anaerobic infections, producing abscesses at different locations and aspiration pneumonia among other serious conditions [4,5].

Phylogenetically speaking the fusobacteria have become somewhat of a puzzle [6]. Originally classified with *Bacteroides *and other Gram negative anaerobes, their association became conflicting when, after the extensive gene sequencing carried out by the mid 80's, it became clear that *Bacteroides *showed a clear relationship to other aerobic Gram negatives such as *Flavobacterium *or *Cytophaga *[7-9] while on the grounds of the 16S rRNA sequence *Fusobacterium *appeared as a separate cluster only distantly associated to the low G+C Gram positives [10,11]. However, this association is methodology sensitive, and different algorithms or genes associate them with other groups such as the Proteobacteria, the Cyanobacteria, the Thermotogales, or within the Firmicutes (see for example [12-14]).

The publication of the *Fusobacterium nucleatum *genome [3] did not solve the problem since although most BLAST top-hits appeared as *Clostridium *species (low G+C Gram positives) genomic analysis showed also a strong proximity to Proteobacteria. Based on the ERGO chromosomal clustering tool, *F. nucleatum *had more "clusters" of genes with the same gene order in common with *Escherichia coli *than with *Enterococcus *or *Staphylococcus*, although less than with *Clostridium *or *Bacillus *[3]. As expected, most elements typical of a Gram negative cell wall were found in the genome including porins, outer membrane transport systems, lipid A synthesis pathways and LPS core compounds. It may be argued that the Gram negative cell wall is the ancestral situation and the Gram positives have lost the outer membrane. However, this scenario requires a remarkable stability in the components of the fusobacterial cell wall to remain so similar to other distant bacterial phyla [15]. On the other hand, there is the possibility that large portions of the fusobacterial genome could be the result of horizontal gene transfer (HGT). The oral cavity environment where *F. nucleatum *thrives is an ecosystem with a large bacterial biodiversity. In a recent survey using 16S rDNA sequences from sub gingival plaque samples, 347 species or phylogroups were identified, and the best estimate of the total species diversity in the oral cavity is approximately 500 species [16]. These 347 species belonged to 9 different bacterial taxa and *F. nucleatum *interacts with a great deal of them, because it plays a crucial "bridge" role between early and late colonizers of the tooth surface [17] and forms carbohydrate-mediated coaggregations with other species [18-21]. Because of the many species with which *F. nucleatum *interacts and aggregates (including spirochaetes, proteobacteria, bacteroidetes, firmicutes, and even fungi) there is a great potential for HGT.

We have reanalysed the fully sequenced genome of *F. nucleatum*, using a variety of bioinformatics tools, in an attempt to clarify the phylogenetic position of the Fusobacteria and the relative contributions of vertical descent and horizontal transfer in shaping the genome of this highly specialized organism. In addition, our study aims at providing material for further discussions on evolution of the gram-negative cell wall, and on the evolution of bacterial communities in micro-environments.

## Results and Discussion

### Phylogenetic position of core fusobacterial genes

**Figure 1:**
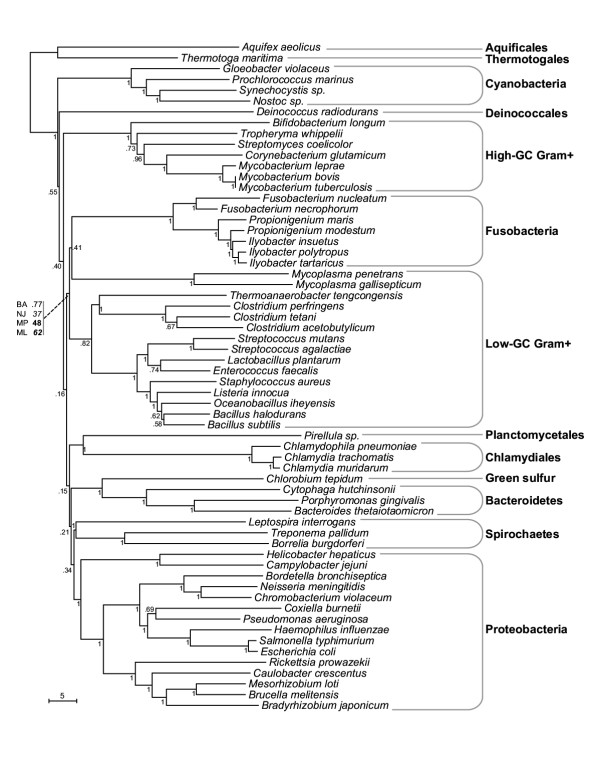
**Phylogenetic tree (Bayesian method) using the combined sequence of the 16S-23S rRNA of representative bacterial species. **The Fusobacteria are a coherent and taxonomically independent group, that branches out at the base of the lineage leading to Firmicutes. Numbers represent bootstrap values. In the case of the branching of Fusobacteria/Firmicutes, the numbers represent the values obtained by four different methods: BA: Bayesian; NJ: Neighbor-joining; MP: Parsimony; ML: Maximum likelihood.

It is generally assumed that in every genome there is, at least, a basic core of genes that are inherited vertically and may be used to infer relationships among prokaryotes [22]. Although most often the relationships obtained with the core genes are consistent with that of the 16S rRNA gene we have extended this type of analysis to include as many genes as possible. Firstly, a Bayesian tree using the combined 16S-23S rRNA sequences was constructed (Figure 1). A neighbor-joining tree based on the concatenated alignments from 44 ribosomal proteins gave a similar result [see additional file 1]. In both cases, the fusobacteria appear as a clearly defined and distinct group that branches out at the base of the Firmicutes but as an independent phylum. Finally, the 23 proteins conserved across all sequenced Bacteria were selected [23] and trees were constructed based on their sequences. Many of them gave results consistent with the previous two trees [see additional file 1]. However, some typical core genes hinted of a mixed ancestry. The ribosome-associated protein prlA, for example, produced a tree that associated *Fusobacterium *with the cyanobacteria and the elongation factor *tufA *with the proteobacteria. Other cases are also unclear. DNA pol III is a complex holoenzyme formed by 10 subunits in *E. coli *[24]. Interestingly, subunits α and β of the polymerase III seem related to Firmicutes while the gene for subunits γ and τ to *Thermotoga *and *Aquifex*. The RNA-directed DNA polymerase and a RNA helicase seem related to archaeal counterparts. In *Clostridium*, for example, to which most informational genes of *Fusobacterium *have a best match, all subunits of both polymerases cluster clearly into the Firmicutes (data not shown). Summarizing, the fusobacteria appear as an independent taxon with remote relatedness to the Firmicutes. Although the phylogenetic signal for many genes was considerably weak, the rRNA genes and the ribosomal proteins were very congruent and therefore their trees (Figure 1 and additional file 1) are likely to represent a reliable phylogenetic reconstruction of the core genome. Heretofore we will refer to this affiliation as ribosomal phylogeny and consider it as the reference for vertically inherited genes. We have assumed that genes showing a close relationship to other bacterial taxa (including the Firmicutes) are possible candidates for HGT origin, particularly when a close association has been proved by more than one approach.

### GC-skew plots

**Figure 2:**
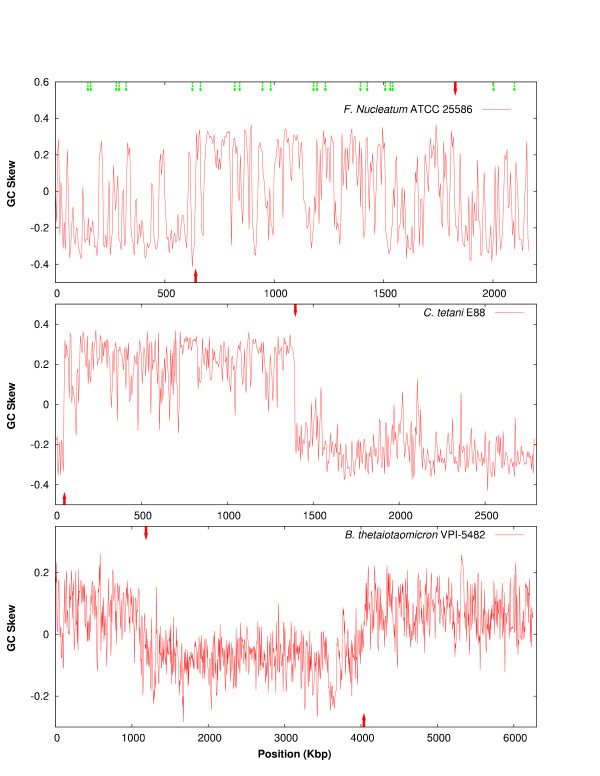
**(G-C)/(G+C) values (GC-skew) plotted every 5000 bp for *Fusobacterium nucleatum*, the low-GC Gram positive *Clostridium tetani *and the bacteroidete *Bacteroides thetaiotaomicron*. **Red wide arrows represent replication origin (bottom) and terminus (top). Orange thin arrows indicate the 36 "clusters" of four or more contiguous genes that are potentially transferred from species outside the Firmicutes. Note that some of *F. nucleatum *shifts in GC skew coincide with putative HGT regions.

Figure 2 shows the GC-skew plot for *F. nucleatum *compared to *Clostridium tetani *(the sequenced species to which it shows the highest number of homologous genes) and *Bacteroides thetaiotaomicron *(the Gram-negative species to which it shows the highest number of homologs). Due to differences in mutational biases between the leading and lagging strands, it is common to find the GC skew value (G-C/G+C) with opposite signs on each replichore, the change in sign indicating the origin or terminus of DNA replication [25,26]. This skew is independent of GC content [27]. As the figure shows, both the Gram-positive and Gram-negative bacteria which appear to have the most similar gene content to *F. nucleatum *display a standard plot, with mainly positive values over the right replichore and negative values on the left replichore. *Fusobacterium*, however, does not show a clear pattern, with constant shifts in GC-skew values across the genome. This situation could be caused by horizontal gene transfer (HGT) incorporating xenologous sequences with a different GC-skew across the recipient chromosome, distorting a clear-cut plot. In favour of this, many GC skew oscillations coincide with clusters of putative xenologous origin (see thin arrows in Figure 2, top). It is interesting to note that an oscillating GC skew plot is also observed in other genomes that appear to have undergone massive HGT episodes (see, for example [28]).

The observed GC-skew could also arise from chromosomal inversions (see, for example, the genome of *Yersinia pestis *-[29]). However, *F. nucleatum *should have undergone massive events of genomic scrambling to account for the effect, including numerous non-symmetric inversions around the replication origin and terminus, which are rarely observed [30,31] and are assumed to be detrimental [32]. Moreover, homologous genes present in the long DNA fragments sequenced in the close relative *F. nucleatum *subsp *vincentii *[33] show an almost perfect sinteny: In all 6 sequenced segments larger than 30 kb in *vincentii*, gene order was conserved without a single chromosomal inversion (data not shown). Although other related genomes are not available for comparison and the potential inversions could have happened prior to the split of both subspecies, the suggestion is that the oscillating GC-skew plot is not due to multiple inversions. Finally, the GC-skew plot of *F. nucleatum *could be partly due to multiple replication origins constantly shifting the values, but this situation has not been observed in any bacterial species.

### Genome sequence similarity analysis

A sequence similarity search performed by BLASTP [34] against the whole available database reveals homology to over 150 bacterial and archaeal species. More than a quarter of the genes had no significant hit or a hit to a eukaryotic species. 64.6% of the hits went to Firmicutes species and 35.4% to other bacterial species (Table 1). These results seem congruent with the ribosomal phylogeny. However, from the hits to Firmicutes, 267 ORFs (representing 12.9% of the total genome) had a hit in only one genus within this bacterial group together with hits in another taxa, a feature suggestive of HGT to or from these bacteria, that are very numerous and diverse in the dental plaque [16].

**Table 1:** General function of *F. nucleatum *genes, divided by group of best BLAST hit^1^.

**Function Category**	**Archaea**	**CFB group**	**Low GC Gram pos**	**α, β, γ Proteo**	**δ, ε Proteo**	**Other eubact**	**Spiro-chaetes**	**Eukarya/No hit**	**Total**
Aa biosynthesis	2 (6.5,3.9)		22 (71,2.3)	3 (9.7,2.0)	2 (6.5,1.9)	1 (3.2,1.4)		1 (3.2,0.2)	**31**
Cofactors and carriers biosynth.	1 (1.4,2.0)		59 (83,6.2)	4 (5.6,2.6)	2 (2.8,1.9)	2 (2.8,2.7)	2 (2.8,2.2)	1 (1.4,0.2)	**71**
Cell envelope	1 (0.6,2.0)	6 (3.6,11.3)	45 (27.1,4.7)	24 (14.5,16)	12 (7.2,11.4)	7 (4.2,9.5)	12 (7.2,13.3)	59 (35.5,10)	**166**
Cellular processes	2 (3.1,3.9)	2 (3.1,3.8)	35 (54.7,3.7)	4 (6.2,2.6)	2 (3.1,1.9)		2 (3.1,2.2)	17 (26.6,2.9)	**64**
Central intermed. metab.	3 (7.0,5.9)	3 (7.0,5.7)	24 (55.8,2.5)	6 (14.0,4.0)	2 (4.7,1.9)		3 (7.0,3.3)	2 (4.7,0.3)	**43**
DNA metab.	4 (5.5,7.8)		45 (61.6,4.7)	5 (6.8,3.3)	3 (4.1,2.9)	4 (5.5,5.4)	1 (1.4,1.1)	11 (15.1,1.9)	**73**
Energy metab.	1 (0.9,2.0)	9 (7.8,17.0)	82 (70.7,8.6)	6 (5.2,4.0)	6 (5.2,5.7)	4 (3.4,5.4)	3 (2.6,3.3)	5 (4.3,0.9)	**116**
Lipid metab.			21 (75.0,2.2)	3 (10.7,2.0)	3 (10.7,2.9)			1 (3.6,0.2)	**28**
Hypothetical prots.		3 (2.1,5.7)	43 (29.9,4.5)	4 (2.8,2.6)	2 (1.4,1.9)	3 (2.1,4.1)	9 (6.2,10.0)	80 (55.6,14)	**144**
Other categories		1 (5.3,1.9)	15 (79,1.6)	1 (5.3,0.7)	1 (5.3,1.0)			1 (5.3,0.2)	**19**
Protein fate		4 (7.0,7.5)	33 (58,3.5)	7 (12,4.6)	4 (7.0,3.8)	4 (7.0,5.4)	1 (1.8,1.1)	4 (7.0,0.7)	**57**
Protein synthesis	1 (0.9,2.0)	1 (0.9,1.9)	88 (78,9.2)	5 (4.4,3.3)	7 (6.2,6.7)	8 (7,10.8)		3 (2.7,0.5)	**113**
Nucleotides metab.	1 (2.9,2.0)	1 (2.9,1.9)	28 (80,2.9)	2 (5.7,1.3)	1 (2.9,1.0)	2 (5.7,2.7)			**35**
Regulat. functions	1 (2.0,2.0)	2 (3.9,3.8)	29 (57,3.0)	3 (5.9,2.0)	3 (5.9,2.9)	2 (3.9,2.7)	5 (9.8,5.6)	6 (11.8,1)	**51**
Signal transduction			3 (60.0,0.3)					2 (40,0.3)	**5**
Transcription	1 (5.0,2.0)	1 (5.0,1.9)	15 (75,1.6)			1 (5.0,1.4)	1 (5.0,1.1)	1 (5.0,0.2)	**20**
Transport/binding proteins	11 (5.7,21.6)	2 (1.0,3.8)	91 (47.4,9.5)	23 (12,15.2)	15 (7.8,14.3)	14 (7.3,18.9)	18 (9.4,20.0)	18 (9.4,3.1)	**192**
Unclassified	11 (7.0,21.6)	3 (1.9,5.7)	72 (45.6,7.5)	16 (10,10.6)	9 (5.7,8.6)	1 (0.6,1.4)	6 (3.8,6.7)	40 (25.3,6.8)	**158**
Unknown function	3 (3.4,5.9)	3 (3.4,5.7)	50 (57.5,5.2)	4 (4.6,2.6)	10 (11.5,9.5)	5 (5.7,6.8)	3 (3.4,3.3)	9 (10.3,1.5)	**87**
Hipothetical function	8 (1.3,15.7)	12 (2.0,22.6)	155 (26.1,16.2)	31 (5.2,20.5)	21 (3.5,20.0)	16 (2.7,21.6)	24 (4.0,26.7)	327 (55,55.6)	**594**
**Total**	**51**	**53**	**955**	**151**	**105**	**74**	**90**	**588**	**2067**

^1 ^First number between brackets indicates the percentage of genes with a best match in a given taxon that have the function indicated on the row heading. Second number between brackets indicate the percentage of genes with a given function that have a best match in the taxon indicated on the column heading.

**Figure 3:**
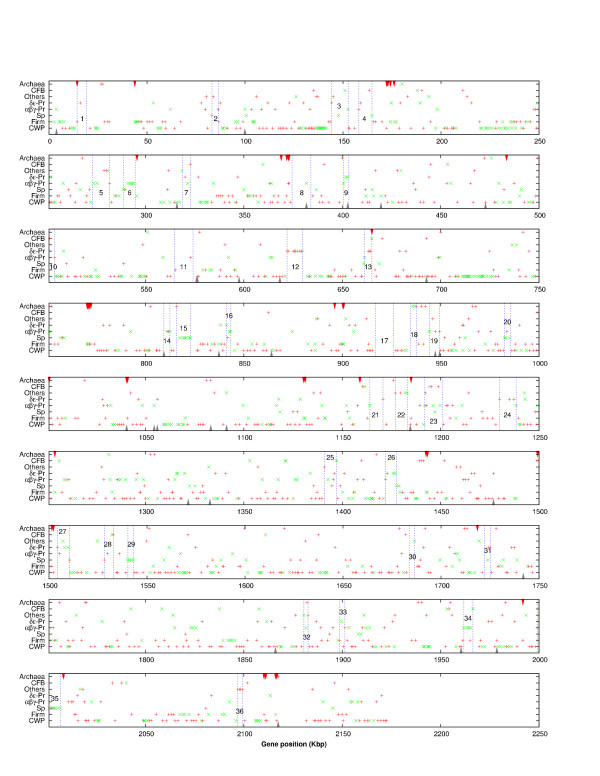
**Gene-position plot with a reconstruction of vertical descent and potentially-transferred genes across *F. nucleatum *genome. **Plus signs indicate genes whose phylogenetic affiliation to a certain group on the left is supported by BLAST analysis only. Crosses indicate genes whose phylogenetic origin is supported by BLAST and by one or two other methods (phylogenetic tree reconstruction and gene order conservation). Thirty-six clusters are indicated containing four or more consecutive genes that appear to have a xenologous origin (i.e. a phylogenetic affiliation outside the Firmicutes). Details of these clusters are explained in Table 2. Arrowheads at the top indicate the position of transposases. Arrowheads at the bottom indicate position of phage-related genes. Plus signs and crosses indicate potential transfers to/from Firmicutes (Firm), Spirochaetes (Sp), alpha-beta-gamma Proteobacteria (αβγ-Pr), delta-epsilon Proteobacteria (δε-Pr), Cytophaga-Flexibacter-Bacteroides Group (CFB), other bacterial groups (Others), Archaea, or genes that are consistent with the phylogeny (CWP) shown in Figure 1 and additional file 1.

**Figure 4:**
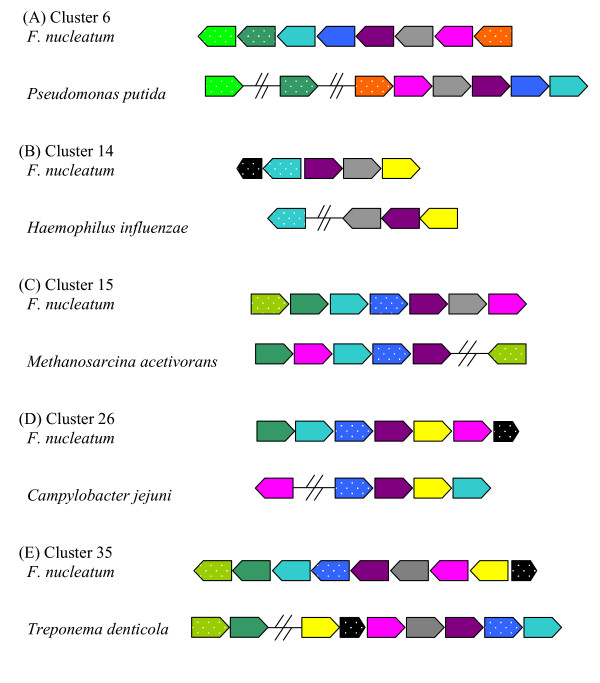
**Gene order conservation in some representative cases of potentially-transferred clusters. **Homologous genes are shown with the same colour in *F. nucleatum *and the species with which it is compared. Small black boxes represent short orphan genes. Non-contiguous genes are separated by an interrupted line. Genes are not drawn to scale.

When the top hits to the different groups are plotted, the matches outside the Firmicutes are scattered along the *Fusobacterium *chromosome and, in many cases, they are clustered (Figure 3). There are 36 cases of clusters of 4 or more contiguous genes that have a best hit outside the Firmicutes, many of which still preserve some gene order compared to their phylogenetically unrelated counterparts. Five of these cases are shown in more detail in Figure 4. Gene arrangement conservation with distantly related groups is a strong indication of HGT events.

It is interesting to note that there are 40 transposase ORFs in the *F. nucleatum *genome and 73 assignments of possible IS elements [3]. Thirty-four of the transposase sequences are at the flanks of putative transferred genes, whereas 6 were between core genes (Figure 3). There are also two integrase genes, both at the edge of putative HGTs. In addition, Kapatral and collaborators [3] described that active and remnant IS-elements are flanking many genes with high similarity to proteobacteria. Among these there are outer membrane proteins, hemolysin precursors and activators, pyrophosphate synthesis genes and others. Another possibility for the insertion of xenologous sequences would be through the action of bacteriophages. In *F. nucleatum*, 31 genes were found to have homologs in phage regions of other bacteria (Figure 3) and 13 on plasmids. Small cryptic plasmids containing mobile elements are frequently found in *F. nucleatum *strains [35]. In addition, six phage contigs encoding 110 ORFs have been identified in its sister subspecies *vincentii*. In this bacterium, the phage genes have homology to Gram positive and Gram negative phages, with an average GC content of 28% and a similar codon usage to the chromosome [33]. Thus, it is possible that an old phage infection is partly responsible for the mosaic genome of *F. nucleatum*. For example, a region with 6 ORFs presents homology to the proteobacterial bacteriophage P2, a phage that has been shown to be responsible for HGT episodes in some *E. coli *strains [36].

Looking into more detail at the gene clusters with a best hit outside Firmicutes it was found that many genes were involved in typical gram-negative features, mainly membrane-associated functions (Table 2). For example, the segment of genes FN1893-FN1897 includes 3 homologs found in *Salmonella typhimurium*, coding for surface-exposed virulence proteins and a membrane-associated gene involved in D-ribose transport. Twenty-eight of the 36 clusters of 4 or more contiguous genes had a function related to the outer membrane, the periplasm or pathogenecity typical of proteobacteria, CFB group species or spirochaetes. Although this would have to be expected since those organisms posses also Gram negative cell walls, the similarity was always to the same groups and much higher than what would be expected based on a distant common origin of the corresponding Gram negative feature. The conservation of gene order and relatively high similarity to groups present in the dental plaque (see below) also hints at a secondary acquisition by HGT. Therefore, the interpretation of *F. nucleatum *as a gram-positive bacterium with gram-negative clothing [6] appears quite realistic, with the xenologous sequences being especially relevant in membrane-associated functions associated to a gram-negative cell wall.

**Table 2:** Clusters of 4 or more consecutive genes with a best match outside the Firmicutes^5^.

#	Putative function	Observations^1^	Sequence similarity^2^	Genes	Sinteny^3^
1	Transposase + 4 hypothetical proteins of similar sequence	Flanked by 3 short orphans^4 ^One of proteins is a short ORF	24–32	FN1511 to FN1515	
2	KDO (LPS core synthesis) + endonuclease and DNA pol III	Includes a short orphan	31–58	FN1561 to FN1576	
3	Peptide ABC transporter	It includes two long (>1500 bp) hypothetical proteins	30–56	FN1650 to FN1656	
4	sysnthesis of LPS (O chain) + phosphatidylcholine synthesis	Split by a hypothetical protein and 3 short ORFs	37–61	FN1661 to FN1668	
5	carbohydrate trasnport-pot operon (periplasmic binding prot dependent transport)	Split by long spacer	22–55	FN1792 to FN1800	*Thermotoga maritima*
6	periplasmic binding protein dependent cation (Mn2+, Zn2+) transport	posibly Co2+ Flanked by transposase and archaeal best-match ORF	24–56	FN1807 to FN1814	*Pseudomonas putida*
7	DNA pol III gamma and tau subunits and TonB OM export system	Flanked by hypothetical orphans	25–36	FN1830 to FN1834	*Helicobacter pylori*
8	Periplasmic amilase and ribose ABC trasnporter	Short orphan in the middle	23–32	FN1893 to FN1897	
9	LPS synthesis and/or decoration and outer membarne stabilization	Flanked by 3528 bp hypothet. protein with eukaryotic best-match followed by long spacer	25–77	FN1908 to FN1911	*Geobacter sulfurreducens*
10	capsule biosynthesis	Includes 2 short ORFs (possible HIPA pseudogenes)	23–46	FN1997 to FN2003	*Bordetella bronchiseptica Yersinia pestis*
11	Slow porin homologous to OmpA (*Bacteroides*) or Opr (*Pseudomonas*)	Split by a long spacer with some homology to membrane proteins. Includes 2 short ORF	23–49	FN2056 to FN2062	
12	Hypothetical exported 24-amino acid repeat protein	Includes 4 short ORFs (one of them with homology to subunit δ of DNA Polym. III)	34–45	FN2110 to FN2122	
13	24 aa repeat protein like in cluster 23	Protein match to *Helycobacter hepaticus*	31–53	FN0023 to FN0028	
14	Endonuclease + 3 genes implicated in porfirinic siderophore synthesis	Flanked by short orphan	24–65	FN0185 to FN0188	*Haemophilus influenzae*
15	DNA helicase + peptide transporters	High gene order conservation in an archaeal species	28–42	FN0191 to FN0197	*Methanosarcina acetivorans*
16	Sugar ABC transporter	Short spacers/overlapping genes	31–48	FN0217 to FN0220	*Escherichia coli*
17	Large cluster of hemolysin/ hemagglutinin containing hemagglutinin *FhaB*	Largest bacterial protein. Some degraded hemolysin copies found throughout genome	23–26	FN0290 to FN0293	*Escherichia coli*
18	ABC iron/haemin transporter with periplasmic binding protein	Flanked by long spacer	27–47	FN0300 to FN0303	*Methanosarcina acetivorans*
19	Periplasmic binding protein dependent iron transport system	Physically linked to other iron transport genes of Gram positive and Archaeal match	34–49	FN0309 to FN0312	*Bordetella bronchiseptica*
20	NA+/H+ antiporter + 3 genes of unknown function	Split by a tRNA gene. Includes 2 short orphans	33–53	FN0350 to FN0354	*Treponema denticola*
21	Two clusters of genes implicated in drug efflux (detoxification) extrusion out of OM	Flanked by two orphans of 402 and 618 bp	21–37	FN0515 to FN0519	*Vibrio cholerae*
22	Mixed functions cluster		30–44	FN0524 to FN0527	
23	LPS synthesis and/or decoration and outer membarne stabilization	Includes recA and recX proteins with best match to *Caulobacter *and *Vibrio*	29–100	FN0538 to FN0548	*Haemophilus ducreyi*
24	Structural lipoprotein with release and mureine anchoring components	Flanked by short ORF	30–46	FN0579 to FN0582	*Helicobacter hepaticus*
25	Membrane-related functions + Fe-S oxidoreductase	Includes a short hypothetical protein with biased codon use	32–55	FN0734 to FN0739	
26	Haemin uptake with periplasmic binding protein iron acquisition	Haemin genes tightly-linked, probable operon	24–59	FN0766 to FN0771	*Campylobacter jejuni*
27	Biotin biosynthesis	Most spacers are short, possible cotranscription	31–55	FN0846 to FN0852	*Campylobacter jejuni*
28	Hydrolase + protease + aromatic compound synthesis	Mixed function cluster	30–47	FN0869 to FN0873	
29	Iron ABC transporter	Flanked by a short orphan with biased codon usage	45–71	FN0879 to FN0882	*Treponema denticola*
30	Membrane proteins	1^st ^and 2^nd ^genes probably permeases	22–37	FN1030 to FN1033	*Photorhabdus luminescens*
31	Lipase B componet of type II secretion system + 24 aa repeat protein+ bacterioferritin	All proteins of short length	26–34	FN1075 to FN1079	
32	KDO (cetodeoxyoctulonic acid biosynthetic operon)	KDO is a component of LPS core in Fusobacterium and many Gram negatives.	31–100	FN1221 to FN1224	
33	Eps synthesis + EpsF (secretion of proteins/large biomolecules)	Possible tandem duplication	30–47	FN1242 to FN1245	*Ralstonia solanacearum*
34	LOS choline decoration + Ton B (biopolymer transport through Outer Membrane)	Includes a short ORF (a degraded copy of a biopolymer transporter)	29–40	FN1306 to FN1312	*Pseudomonas aeruginosa*
35	ABC transporter system	Flanked by short orphan followed by a transposase	30–69	FN1346 to FN1355	*Treponema denticola*
36	ABC amino acid transport system	*liv *G-M operon; biased and homogeneous codon usage	50–62	FN1428 to FN1431	*Bifidobacterium longum*

^1 ^"Split" indicates a cluster separated by a long intergenic spacer, the two parts of the cluster generally coding for different functions.

^2 ^Range of sequence similarity among the genes from each cluster compared to their BLAST top hits.

^3 ^Representative species with similar gene order.

^4 ^An "orphan" gene is defined as an ORF with an unknown function and no BLAST similarity in the current database. Short orphans (<500 bp) are likely to be pseudogene remnants or other non-functional regions (Mira *et al*. 2002, Davies *et al*. 2004).

^5 ^Only protein coding genes are included in the analysis.

### Phylogenetic, gene-order and compositional analyses

BLAST analysis has the advantage of giving a closest similarity match for almost every gene. It is however a crude method that can give as the top sequence similarity hit a species that is not the closest from a phylogenetic point of view [37] and it could also be much influenced by the undersampling of certain poorly-sequenced groups, such as the Fusobacteria. For example, when the BLAST top 10 best matches are considered, less than 70% of the hits fall on the same bacterial taxa as the top hit. In top hits to archaea, a domain from which fewer sequences are available, 58% of the top ten best matches hit groups other than Archaea. Since we used the top hit to designate potential phylogenetic origin, some degree of inaccuracy is expected. Thus, to complement the BLAST analysis we used a phylogenetic and a gene-order analysis, indicating in Figure 3 whether their results do or do not support the BLAST results. In the phylogenetic analysis, trees were constructed based on the sequence of each individual gene with sufficient homologs in the database (see experimental procedures section for details). Over 1200 trees were generated and analysed to detect a phylogeny either congruent with the ribosomal one, or suggestive of HGT. Almost two thirds of the trees could be resolved, and corroborated the high degree of gene transfer, with at least 25% of the genome being of xenologous origin (Table 3). Only 8.4% were consistent with the ribosomal phylogeny, and over 25% indicated a potential HGT to or from Firmicutes species. Part of the latter could also be due to multiple losses of the genes in most Firmicutes genera. However, it is not unreasonable to think that they could have been transferred between Fusobacteria and typical Firmicutes (particularly *clostridiales *and *streptococci*), which share the mouth and dental plaque ecosystem [16]. The phylogenetic analysis method, therefore, suggests a 25–50% of gene transfer. Although only trees with a bootstrap value over 500 were considered (see methods section), these numbers must be taken with caution. Given the distant relationship of *F. nucleatum *with most sequenced genomes, a weak phylogenetic signal may remain for many trees. The branching pattern in trees is also influenced by other variables like different rates of evolution for different genes, method of alignment or number of species included. Like in the sequence similarity method, the data presented are inferences based upon the data, and the limitations of each method should be kept in mind.

**Table 3:** Percentage of *F. nucleatum *ORFs classified by the taxa of potential origin.

	Sequence similarity method (BLAST)	Phylogenetic trees method	Gene order conservation
Number of genes analyzed	2067	1236	738

Root of Firmicutes^1^	33.28 %	8.41 %	35.1 %
Inside Firmicutes^2^	12.92 %	25.8 %	15.45 %
CFB group	2.56 %	2.27 %	4.06 %
α, β, γ Proteobacteria	7.34 %	10.3 %	21.0 %
δ, ε Proteobacteria	5.07 %	3.4 %	5.7 %
Spirochaetes	4.35 %	4.32	6.37 %
Other eubacteria	3.58 %	4.53	4.2 %
Archaea	2.46 %	1.13 %	0.95 %
No hit, hit to eukaryotes, uncertain/unresolved	28.4 %	38.7 %	7.45 %

^1 ^Genes consistent with the 16S-23S and ribosomal proteins phylogeny.

^2 ^It indicates possible HGT to/from Firmicutes.

Another method used was based on the conservation of gene order among certain gene clusters, a character that can be used in phylogenetic reconstructions [38,39]. Only 738 *F. nucleatum *protein-coding genes belonged to clusters of 2 or more genes that had some order conservation in other bacteria. From these, 35% had the same order as most Firmicutes (Table 3), suggesting vertical inheritance. Over 15% of the genes belonged to clusters whose gene order was more consistent with HGT from this group (i.e. same order as only one of the Firmicutes genomes). The extent of HGT from Firmicutes could be overestimated if the genes are ancestral but subsequently lost in most Gram-positive lineages. This being the case, the addition of vertically-inherited genes and genes inside Firmicutes in Table 3 would indicate an upper limit of genes consistent with the ribosomal phylogeny. Even if HGT from Firmicutes is not considered, 42% of the genes were assigned as HGT from other bacterial taxa based on the gene-order method. These dramatic figures suggest again that the genome of *F. nucleatum *could be an amalgamation of genes from different groups, particularly those of species that inhabit mammalian hosts in general and the mouth niche in particular. A summary figure showing the outcome of the three methods is published as supplementary material [see additional file 1]. The discrepancies between the three methods can be partly influenced by the different sample sizes used (Table 3). In addition, it must be noted that most of the discrepancy appears in the Phylogenetic Trees method, where a very low percentage of vertical inheritance was detected. In this analysis, over 38% of the trees were unresolved, introducing an important degree of variation. It is therefore possible that many of the genes giving uncertain phylogenies are consistent with vertical inheritance, but the phylogenetic signal is too weak to give a clear-cut tree. The gene-order method could give higher numbers of horizontal transfers if operons are more likely to be transferred than single genes [40]. Thus, all three methods have its limitations, and although the importance of HGT is clear, the numbers obtained may be subject to certain bias imposed by the methodology [41].

Deviations from genomic GC content and codon usage have been used to infer potential gene transfers across bacteria [42,43]. However, only 40 genes with significantly extraneous DNA composition were found in *F. nucleatum *[44] suggesting that many transfers could come from low-GC species or that many of the transfers occurred long ago, allowing the xenologous genes to ameliorate and homogenize its characteristics with those of the recipient genome [45]. In addition, the extremely low GC content of *F. nucleatum *could make this method less discriminatory [46,47]. A few potential transfers were identified this way, including a cluster spanning two iron-sulphur binding proteins and two arsenic pump-driving ATPases. Another interesting case was a glutamate fermentation cluster with closest similarity and gene order conservation to the clostridial species *Acidaminococcus fermentans*. This represents a typical case of potential HGT from the Firmicutes that could be masked in a BLAST analysis as a vertically inherited cluster. As the tree and gene-order methods show, the amount of HGT from/to the Firmicutes species could be as high as 15–25%, assuming that the percentages are maintained among the genes that we could not analyse because the trees were unresolved or because they were not part of conserved-order clusters.

### Chimeric enzymes and operons

To explore the possibility that the chimeric nature of *Fusobacterium *may apply not only to its genome but also to some of its metabolic pathways and enzymes, some specific cases were looked at in more detail. A potential example includes the RNA polymerase, where the β' subunit has a best BLAST hit to spirochaetes as well as the RNA polymerase sigma-E factor. This is confirmed by comparative analysis of domain architecture across bacteria [48]. An interesting instance is given by the phenylalanyl-tRNA synthetase, in which the α and β chains have a *Clostridium *and *Geobacter *(delta-proteobacteria) best sequence similarity match, respectively. The tree analysis confirms that the β chain is likely to have a proteobacterial origin. Interestingly, although the β chain is located in a proteobacterial cluster (at the edge of cluster 12), it is contiguous to the Firmicutes related α chain gene, separated by a very short spacer without a promoter. This exemplifies how selection may have put together two functionally related genes, presumably to ease cotranscription, even though their phylogenetic origin appears to be different.

**Figure 5:**
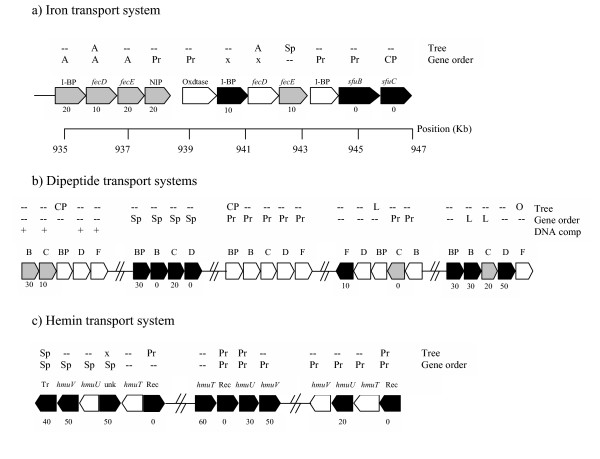
**Chimeric operons (metabolic pathways of putatively mixed origin) in *F. nucleatum*. **Arrowed boxes represent gene orientation, coloured by BLAST top hit. White boxes: top hit to Firmicutes; grey boxes: top hit to Archaeal species; black boxes: top hit in Gram negative species. Numbers below boxes indicate the percent of top ten hits that have matches in Firmicutes. Names above indicate gene names (I-BP: Iron binding protein; NIP: Nitrogenase iron protein; Oxdtase: Oxidoreductase; B, C, D, F: dipeptide permeases B, C, D, F; BP: dipeptide binding protein; Tr: ABC transporter; unk: unknown function gene; Rec: Hemin receptor). Best match taxa by the phylogenetic tree and gene order methods are also indicated (A: Archaea; Pr: Proteobacteria; Sp: Spirochaetes; CP: consistent with (ribosomal) phylogeny; O: other eubacteria; x: unresolved; --: not analysed. Plus signs indicate unusual DNA composition by the method of García-Vallvé *et al*. 2003.

Another example is given by an iron ABC transporter operon formed by a periplasmic binding protein followed by two iron permeases. A similar structure is repeated two other times in the subsequent genes (Figure 5a), forming a long iron transport system. Remarkably, the first operon is found in identical gene order in the archaeon *Methanosarcina acetivorans*, to which it presents the highest sequence similarity, whereas the second and third operon appear to be a blend of genes with relatedness to firmicutes, proteobacteria, spirochaetes and *Thermotoga*. These genes are present in many Gram negatives including *Helicobacter *and other Proteobacteria [49,50]. Thus, assuming that some of these genes have a xenologous origin, they must have been selected to occupy a precise gene order to maximise its function within the iron transport system. Another fascinating case of a potential chimeric gene system is that of transport of dipeptides (Figure 5b). There are as many as five dipeptide transport operons in *F. nucleatum*, this time dispersed along the genome. Although one of the sets has best matches to Firmicutes species, another one appears to be of spirochaete source (also present in the same gene order in *Methanosarcina*), whereas the other three are, according to sequence similarity, gene order and tree reconstruction a mixture of genes with archaeal, Firmicutes and proteobacterial origin. A third case can be seen in the three copies of a hemin transport system located away from each other and formed by a hemin receptor and the genes *hmuT*, *hmuU *and *hmuV*. Although the different taxonomical origin analysis methods are not always consistent, two of the hemin operons are probably of proteobacterial origin (Figure 5c). The other one has a closest gene order to the spirochaete *Treponema denticola*, also an inhabitant of the dental plaque, but is absent in its close relative *T. pallidum*, suggesting again that gene transfer is facilitated across the bacteria that occupy this specialised niche.

### Remnants of HGT

An indication of massive gene transfer events comes from looking at intergenic spacer regions of *F. nucleatum*. Although average spacer length in this species is 115 bp, there are many long spacers of 500 bp and higher scattered across the genome. It was found that 21 of these long spacers were located at positions flanked by a "core" gene (that with a low-GC Gram-positive best match) and a potential transferred gene, whereas only 8 appeared between core genes. Since intergenic spacer regions are known to increase in length as a result of genomic rearrangements and pseudogene formation [51], many of these long spacers might be signatures of ancient HGT events. In agreement with this view, another 17 long spacers were located inside gene clusters of a putative Gram negative or archaeal origin. We hypothesize that these long non-coding regions are remnants of transferred genes that were not selected for and have been mostly erased. When DNA sequence similarity searches are done with these long spacers located inside xenologous clusters, some significant matches are found to other regions of the genome. For example, the long spacers inside clusters 4, 8 and 11 all have some sequence similarity (more than 85% sequence identity over 125 bp or more using BLAST analysis, E-value <10^-5^) to one another and to other five long spacers scattered throughout the genome. In all cases except one, these long spacers are flanked by outer membrane proteins of Gram-negative origin, suggesting that they may represent remnants of old membrane-associated genes. A similar case is that of the long spacer located after the hemolysin activator protein precursor (FN1818), which shows high sequence similarity to a hemolysin activator located someplace else in the genome (and to another spacer and a short ORF with unknown function).

**Figure 6:**
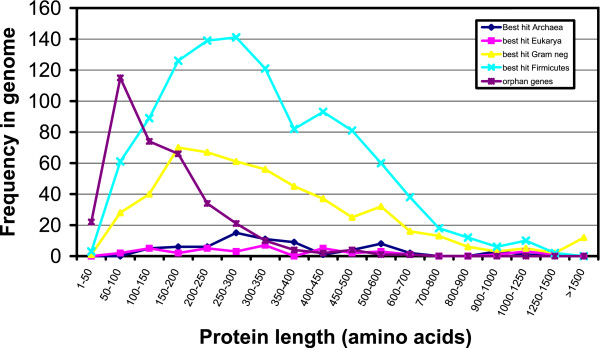
**Length frequency distributions for *F. nucleatum *proteins. **Genes are divided by the group with closest sequence similarity match. ORFs without sequence similarity on the non-redundant NCBI database (orphan genes) are significantly shorter than the rest.

Another potential signature of ancient transfers subsequently erased is the high number of ORFs without a match in the complete, non-redundant NCBI database (including its closely related subspecies *vincentii*), spanning 450 sequences that represent 20% of the genome. On average, these orphan genes are extremely short (440 bp, versus 1040 bp for the rest of genes with a match on the DNA database, see Figure 6), suggesting that they do not represent real genes [52,31]. Overannotation of short ORFs that are not functional is more common on GC-rich genomes due to a lower probability of stop codons [53], but *F. nucleatum *is just 27% G+C. It is therefore possible that many of these short ORFs are eroded pseudogenes or remnants of fragmented genes, as it has been demonstrated in *Rickettsia *[54], where many genes appear to be under low selection coefficients in its intracellular environment. In *Fusobacterium*, it is likely that many transferred genes were not useful and got eliminated, a process known to happen very rapidly [55]. This would explain the high number of short ORFs without significant BLAST matches on the database, as small fragments of genes may have accumulated enough mutations to make them frequently unrecognisable by sequence similarity. For many of these small ORFs with no significant matches, some low sequence similarity is found to gram-negative outer membrane proteins (e.g. *tolA*), glycine permease, periplasmic-like proteins, etc.

In addition, some short ORFs appear to be degraded fragments of bigger genes. For example, there are 3 sequences with similarity to HIPA proteins, one of which is less than half the length of the other two. As it also has a very biased codon usage, it is likely that it represents a degraded remnant of this protein. The 3 copies of integrases scattered across the genome show another case. Two of them are around 900 bp long and have a normal codon usage. The third copy (FN0402) is only 177 bp long, is flanked by a long spacer and has a very skewed codon usage. In general, the codon usage of these orphans is very biased (mean corrected χ^2 ^values of 0.47 versus 0.22 for the rest of the genome). As it is unlikely that all these short ORFs are highly expressed, we believe that this biased codon utilization is reflecting very divergent pseudogene fragments. Thus, the picture that emerges is that of massive gene transfer leaving many non-coding segments that are remnants of unnecessary genes and genomic rearrangements.

## Conclusions

The genome of *F. nucleatum *possesses a remarkable amount of patchiness with any kind of phylogenetic analyses used. This can be said to a certain degree of some other genomes (see for example [56]). One possible explanation for this kind of results is an undersampling of the group considered what gives only very distant and hence uncertain similarities to a variety of prokaryotic groups. This might be the case for part of the *Fusobacterium *genome that gives very weak and uncertain phylogenetic signal. However, the observation that certain genes and operons are shared by distantly related species that inhabit the dental plaque (for example, the spirochaete *T. denticola*, the proteobacteria *Campylobacter *and the CFB *P. gingivalis*) points to HGT as the most likely origin of these genes. Even less apparent, our work suggests multiple episodes of gene transfer to or from phylogenetically-related bacteria, like certain Firmicutes species (such as the cariogenic bacterium *S. mutans *or some Clostridia), that might be confounded with vertically inherited traits.

The origin of the Gram-negative cell wall found in *Fusobacterium *requires special consideration. Some type of Gram-negative cell wall seems to be the default phenotype in Bacteria (see, for example [57]), being found in most deeply branching groups. Moreover, even some deep branches of the Firmicutes contain organisms (such as *Sporomusa *and *Desulfotomaculum*) with Gram-negative cell wall structures [58,59]. On the other hand, it has also been proposed that the Gram-positive cell wall is the default structure [60]. It might be argued that Fusobacterium is a remnant of the ancestral cells predating the bacterial radiation that originated either Gram-positive or Gram-negative cell walls. This is supported by phylogenetic inferences based on conserved indels, which place Fusobacteria at an intermediate position between Gram-positive and Gram-negative taxa [61]. However, in light of our results this explanation does not seem likely. *Fusobacterium *does not show any primitive trait and its outer membrane and transport mechanisms show all the characteristics of any sophisticated Gram-negative cell wall. In addition, many of the outer membrane proteins are closest to specific taxa (mainly to proteobacterial species) and not equally dispersed among species with a Gram-negative cell wall. Thus, many of the genes involved in the construction of the Gram-negative outer membrane have probably been horizontally transferred. The extent of this transfer deserves further examination. If we assume that *Fusobacterium *evolved after the Gram-positive/negative divergence on the low-GC Gram-positive lineage, massive HGT is the most likely explanation for the formation of the outer membrane. On the other end of possible explanations, most genes of the outer membrane would already be present in the common ancestor of fusobacteria and Firmicutes, where a massive loss would be responsible for the differences observed today.

Recently, the idea of gene pools that are characteristic of certain environments has been advanced to explain the large number of common genes among groups of thermoacidophiles distantly related by ribosomal phylogeny [62]. The presence of a common pool of dental plaque genes is not unlikely in light of the results described here. However, the time scale of the adaptation to the latter habitat is much shorter that that of thermoacidophiles and can be probably estimated around the origin of mammals (about 120 million years). Even going backwards to the origin of the vertebrate's intestine it would put the selective pressure for these gene combinations to originate no earlier than 400 Myr ago. Former chimeric genomes have been explained as selected by strong environmental pressure. The case of *Thermotoga *is paradigmatic, a hyperthermophilic bacteria that is assumed to have recruited genes from the archaeal hyperthermophiles to reach its unusual (for bacteria) thermotolerance. Here (as in the case of *Methanosarcina*, a mesophilic anaerobe) there is not such an obvious explanation. *F. nucleatum *natural habitat seems to be the dental plaque of mammals, a rather unique and special environment that probably requires very special features to survive. Strong adhesion mechanisms, such as those found often in the Proteobacteria, probably represent an essential ability for survival in the early stages of plaque formation, particularly for non-motile cells. Also the mucose-associated immune system that prevails in the mouth of mammals could have acted as a strong selective pressure favoring the Gram-negative envelopes that are often less immunogenic and easier to disguise thanks to the LPS polysaccharide O chain [63]. Thus, it is not difficult to envisage that at a point in time, probably associated to the rise of mammals, a strong selective pressure might have existed for a cell with the metabolic apparatus of Clostridia for amino acid fermentation but with the adhesive and immune camouflage paraphernalia of the Proteobacteria. It is remarkable to note that many of the genes that determine the lifestyle of *Fusobacterium *and its interaction with the environment, such as peptide transport systems, cell adhesins and outer membrane components have probably been acquired by gene transfer. It is therefore not only the number of horizontal transfers but also their contribution to niche adaptation that makes the HGT mechanism of dramatic impact on genomes. It is interesting that some of these genes are shared by different organisms inhabiting the dental plaque. From an applied point of view, some of these highly transferred genes are likely to provide a critical advantage in the establishment and adaptation of the bacteria to their niche, and could be used as potential targets for antimicrobial agents.

## Methods

### Phylogenetic trees

#### rRNA and evolutionary conserved proteins trees

The different rRNA and conserved protein data sets were analyzed with Bayesian methods using the program MrBAYES 3 [64]. For the fusion of 16S+23S rRNA sequences, the GTR model with a Γ law (8 rate categories) and a proportion of invariant sites to take among-site rate variation into account was used. A similar procedure was used to construct the trees based on evolutionary conserved proteins (a mixed substitution model and a Γ law with 8 rate categories and a proportion of invariant sites were applied). The evolutionary conserved proteins were defined as those found in all sequenced species of Bacteria and assumed to form part of the minimal genome necessary for life [65,66]. The list was extracted from [23] but removing the genes for which paralogous ORFs were found. In all cases, the Markov chain Monte Carlo searches were run with 4 chains for 1,000,000 generations, with trees being sampled every 100 generations (the first 2,500 trees were discarded as "burnin").

#### Concatenated ribosomal proteins tree

The amino-acid sequences of ribosomal genes S1–S20 and L1–L35, excluding S1, S14, L24, L25, L30, L31, L32 and L33, were retrieved from the KEGG website from a total of 60 different bacteria. The bacteria chosen were all those represented in the KEGG ribosomal genes ortholog table [67], except *Rickettsia prowazekii*, *Rickettsia conorii*, *Wigglesworthia brevipalpis*, and *Buchnera aphidicola*, and with the addition of *Bacteroides thetaiotaomicron *and and *Desulfovibrio vulgaris*. An alignment was generated for each ribosomal gene, using the Clustalw software with default parameters [68]. When two or more paralogs were found in a species, the most divergent of the paralogs was removed from the alignment. A concatenated alignment including the species for which all of the selected ribosomal genes were present was generated. A neighbor-joining tree with 1000 bootstrap replicates was produced from the alignment using Clustalw [68], excluding positions with gaps, and correcting for multiple amino-acid substitutions (Kimura correction). The tree was visualized with NJPLOT [69]. Exclusion of ribosomal proteins was based on the following: S14 has been shown to be subject to horizontal transfer [70], L24 is truncated and split in *Fusobacterium nucleatum*, S1 is absent/truncated in the Mollicutes subgroup of the low-GC gram-positives, L25, L30, L31, L32, L33 contained a high number of paralogs and/or were absent in several key species.

### Methods for detecting HGT

#### Blast method

The protein sequences of *Fusobacterium nucleatum *subsp. *nucleatum *ATCC 25586 were retrieved from . Peptide sequence database of all non-redundant GenBank CDS translations + PDB + SwissProt + PIR was retrieved from . We performed an all against all BLASTP [34] search of each protein in *Fusobacterium nucleatum *subsp. nucleatum ATCC 255586 against peptide sequence database. We then recorded the top hit for each protein sequence with an E-value of 10^-5^, filtering the hits whose sequence identity and length was lower than 30 and 50%, respectively. We categorized all the hits into 8 categories as belonging to the CFB group, Firmicutes bacteria, α,β,γ-Proteobacteria, δ,ε-Proteobacteria, Spirochaetes, other Bacteria, Archaea and Eukaryotes/no hit. Hits to Firmicutes (the group to which *Fusobacterium *appear to be more closely-related) were refined by further BlastP analysis between *F. nucleatum *and the 31 sequenced bacteria available from this group. If the gene had a homolog in only one genus from all the available low-GC gram-positive species, it was considered a HGT event from/to this group. If it was present in more than one genus it was considered vertically inherited and consistent with the ribosomal phylogeny. There were 61 cases of genes found in more than one genera from a single subgroup of this taxon (i.e. present only in the Clostridiales, the Bacillales, the Mollicutes or the Lactobacillales). These can be equally explained by HGT or by common descent and were conservatively assigned to the vertical inherited category.

#### Phylogenetic trees method

For each *F. nucleatum *gene, the protein sequences of up to 50 best blast hits with e-value lower than e^-5 ^were retrieved (the hits were identified by the "Blast method" described above). All sequences were then automatically clustered with the Clustalw alignment tool with default parameters. A neighbor-joining tree with 1000 bootstrap replicates was generated from the resulting alignment, using Clustalw with default parameters. The trees were visualized with NJPLOT [69]. In all cases, the bootstrap values at the nodes chosen for a decision on taxonomical assignment had to be over 500. Assignment of the *F. nucleatum *genes to a taxonomic group was done using the following criteria:

##### Low-GC gram-positives

A *F. nucleatum *gene was determined to originate from the firmicutes if it was found in the tree most closely associated with at least 5 different species from that group, or with at least 3 species from 2 different subgroups (where the subgroups were: mollicutes, bacillales, lactobacillales, clostridiales). If the *F. nucleatum *gene branched at the base of the firmicutes, the gene was assigned as being consistent with phylogeny; otherwise, it was assigned as a potential horizontal gene transfer (HGT) from the firmicutes.

##### Proteobacteria

Same as described above (low-GC gram-positives), the subgroups in this case were:alpha-, beta-, gamma-, gamma-entero-, delta-, and epsilon-proteobacteria. In the case of the proteobacteria, all *F. nucleatum *genes with trees fulfilling this criteria were assigned as HGTs from proteobacteria. Note that a distinction was made between the grouping of the alpha-, beta-, and gamma- proteobacteria, and the grouping of the epsilon- and delta- proteobacteria whenever possible.

##### Archaea

To be assigned as originating from the archaeales, the *F. nucleatum *had to be closest to at least 3 species, and there had to be a clear association between the two groups, i.e. the branches were relatively short, and the tree topology did not resemble a "star phylogeny".

##### High-GC gram-positives, Cyanobacteria, Chlamydiales

To be assigned to these groups, the *F. nucleatum *gene had to be found closest in the tree to at least 3 different species, there had to be a clear association (see above in "Archaeales") and there should have been no obvious evidence of gene transfer from the Fusobacteria. Evidence of transfer from the Fusobacteria would be when, apart from the association to some species of the high-GC gram positives (or Cyanobacteriales, or Chlamydiales), the tree placed the *F. nucleatum *gene in agreement with the accepted species phylogeny (just outside of the firmicutes).

##### Aquifales, Deinococcales

The *F. nucleatum *gene had to be found closest to at least 1 species from that group (Aquifales, or Deinococcales). A clear association was necessary, as well as no evidence of transfer from Fusobacteria (see above).

##### Spirochaetes, CFB group

The *F. nucleatum *gene had to be closest to at least 2 species from that group, or 1 species with a clear association, and no evidence of transfer from Fusobacteria.

##### Unknown

If the tree contained less than four hits other than eukaryotes and other Fusobacteria, the gene was not considered for further analysis. In cases where it was not possible to clearly associate a taxonomic group to the *F. nucleatum *gene, it was then assigned as "unknown/not resolved".

#### Gene-order method

In order to identify clusters of at least two genes with conserved order between *F. nucleatum *and other genomes, all available amino-acid protein sequences sets for all replicons of all published bacterial and archaeal genomes at the time (may 1^st ^2004) were downloaded from the NCBI ftp website . All Orthologs of *F. nucleatum *genes (reciprocal best blast hit) were detected between the two replicons (*F. nucleatum *and replicon X). Clusters of consecutive orthologs were found (consecutive orthologs are determined in terms of the numbered position in both *F. nucleatum *(exactly consecutive) and replicon X (possible gap of 2 genes in between the orthologs), and for each cluster, a score was assigned as follows:

(1-(totalCost/numberOfGenesInCluster))*(1-(deletions/numberOfGenesInCluster)) *Mean(Identity%)*Mean(Length%)*numberOfGenesInCluster/10000

Where "totalCost" was determined by the program derange2 [71,72] with the following command-line: "derange2 -U -L $inputFile 5 1 1 1", i.e. the direction of the gene was ignored, and the cost for an inversion, a transition or translocation within the cluster was the same: 1."Deletions" is the number of "gene gaps" found in replicon X for that cluster, whatever their size, "Mean(Identity%)" is the mean of the %identity of all blast results for the orthologs of the cluster. Mean(Length%) is the mean of the length of all blast results for the orthologs of the cluster, where length is defined as the minimum of length of (blast hit/ length of query sequence) and (length of blast hit/length of subject sequence). Each ortholog in the cluster was assigned the same score, and following completion of the procedure for all replicons in the database, for each *F. nucleatum *gene the orthologs that were part of clusters were ordered by their score, and an excel table was generated for manual investigation. If the gene order of a given gene cluster was not preserved in Firmicutes species but maintained in another procaryotic group, the genes were assigned as HGT from/to the group with the highest score (highest gene-order conservation). If the order was preserved in at least one species from two or more groups of low-GC gram-positives (Clostridiales, Bacillales, Lactobacillales and Mollicutes) the cluster was assumed to be ancestral to the divergence of fusobacteria and Firmicutes, and consistent with the ribosomal phylogeny. If gene order was preserved in one or more species from only one of the low-GC gram-positive groups the cluster was classified as HGT from low-GC gram-positive bacteria.

### GC-skew plots and gene classification

Classical GC-skew plots were done using the formula (G-C)/(G+C) in 5000 bp windows, following Lobry's methods [25,26]. The functional classification of *F. nucleatum *genes by function was based on the TIGR Gene Attribute Annotation [73].

## Authors' contributions

AM carried out the gene-order analysis of gene transfer, and the study of chimeric operons and short ORFs. FRV conceived the study, and FRV and AM drafted the manuscript. RP and BAL did the bioinformatics work. RP and AM carried out the BLAST analysis of gene transfer and made the figures, except the phylogenetic trees. BAL developed the phylogenetic and gene-order method and did the ribosomal proteins tree. DM did all Bayesian trees. All authors read and approved the final manuscript.

## Supplementary Material

Additional File 1 **Supplementary material published as additional information in the manuscript Mira et al. 2004. Evolutionary relationships of *Fusobacterium nucleatum *based on phylogenetic analysis and comparative genomics. **The file contains three additional figures. It is available in pdf format and includes figure legends. Click here for file
