# Supplementary material for: Evolutionary relationships of Fusobacterium nucleatum based on phylogenetic analysis and comparative genomics
Source: BMC Evol Biol. 2004 Nov 26;4:50. doi: 10.1186/1471-2148-4-50 (PMC535925; doi:10.1186/1471-2148-4-50)

Mira et al. 2004. Evolutionary relationships of *Fusobacterium nucleatum* based on phylogenetic analysis and comparative genomics

Supplementary material published as additional information.

Additional Information Figure 1. Concatenated ribosomal proteins sequences tree. Numbers indicate bootstrap values. The Fusobacteria are an independent phylum that branches out at the base of the lineage leading to low-GC Gram positives. When the Rickettsiales and *Buchnera* were included, the delta-proteobacteria *Desulfovibrio vulgaris* appeared outside of the proteobacteria; when no correction was applied, the position of the mollicutes (*Mycoplasmas* and *Ureaplasma*) and *Fusobacterium nucleatum* branches was reversed. To improve visibility, *Deinococcus radiodurans* was chosen as an outgroup, and most bootstrap values (except those with values >.995) were rounded to two decimals.

Additional Information Figure 2. Bayesian trees of some evolutionary conserved proteins (genes dnaN, fusA, nusG, tufA, prlA, infA). In general, the phylogenetic signal was weak, and different trees gave associations with various bacterial phyla.

Additional Information Figure 3. Origin associations of *F. nucleatum* genes by the three methods used for reconstructing phylogenetic reconstruction of each gene. a: sequence similarity by BLAST; b: phylogenetic reconstruction by neighbour-joining trees; c: gene order conservation. Every row in the chromosomal plot represents 420 Kbp.

Additional Figure 1

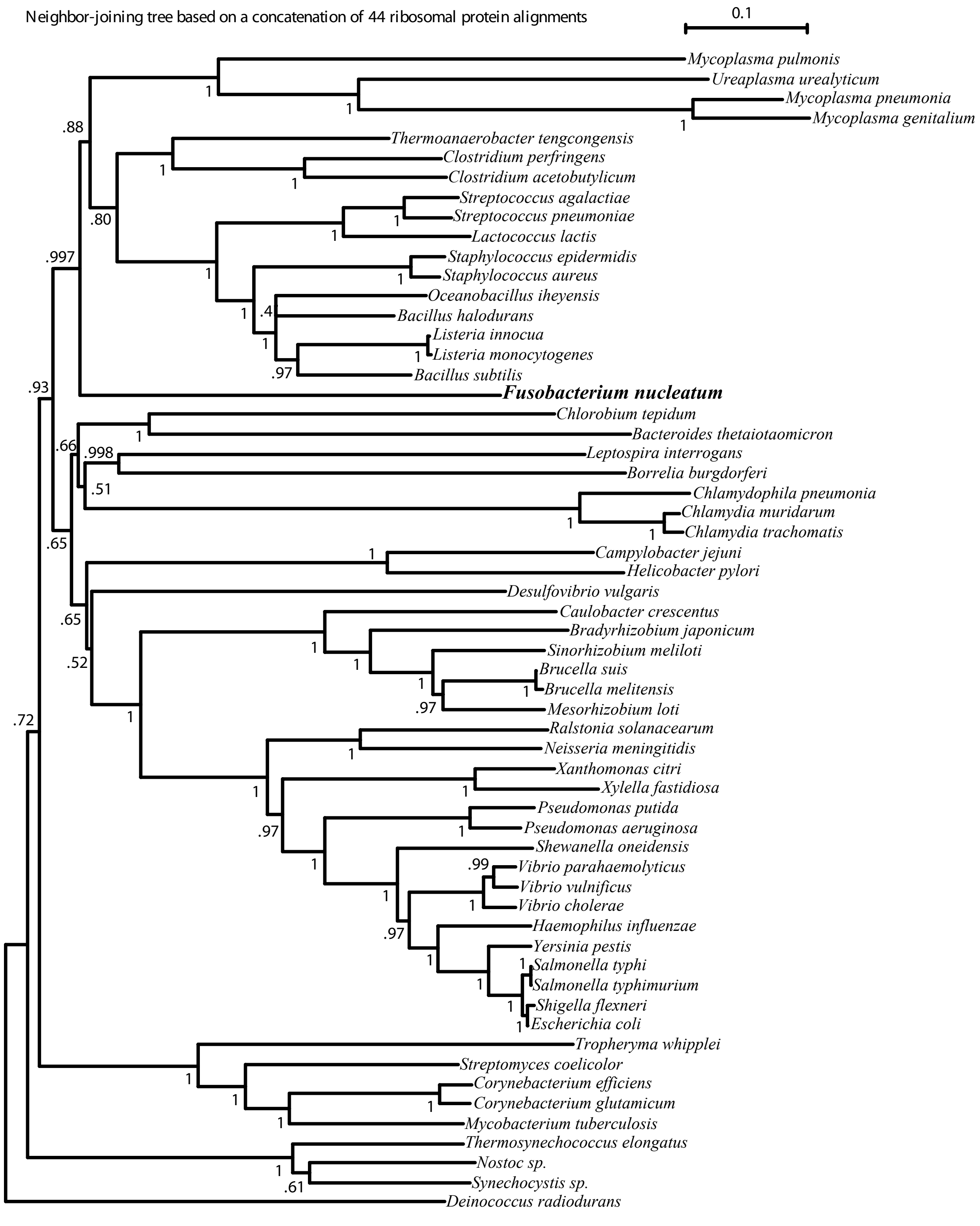

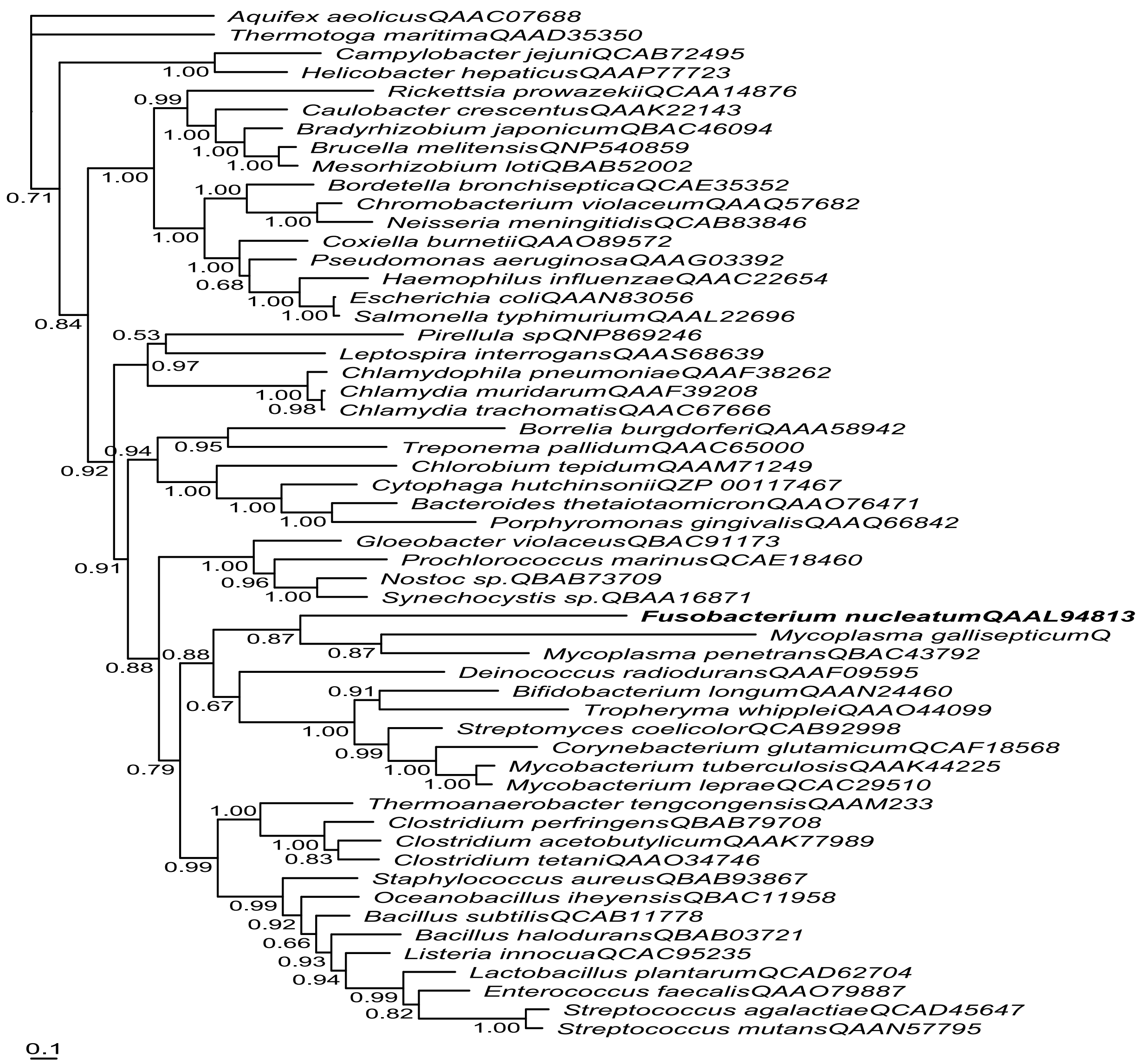
Aquifex aeolicusQAAC07688
Thermotoga maritimaQAAD35350
Campylobacter jejuniQCAB72495
Helicobacter hepaticusQAAP77723
Rickettsia prowazekiiQCAA14876
Caulobacter crescentusQAAK22143
Bradyrhizobium japonicumQBAC46094
Brucella melitensisQNP540859
Mesorhizobium lotiQBAB52002
Bordetella bronchisepticaQCAE35352
Chromobacterium violaceumQAAQ57682
Neisseria meningitidisQCAB83846
Coxiella burnetiiQAAO89572
Pseudomonas aeruginosaQAAG03392
Haemophilus influenzaeQAAC22654
Escherichia coliQAAN83056
Salmonella typhimuriumQAAL22696
Pirellula spQNP869246
Leptospira interrogansQAAS68639
Chlamydophila pneumoniaeQAAF38262
Chlamydia muridarumQAAF39208
Chlamydia trachomatisQAAC67666
Borrelia burgdorferiQAAA58942
Treponema pallidumQAAC65000
Chlorobium tepidumQAAM71249
Cytophaga hutchinsoniiQZP 00117467
Bacteroides thetaiotaomicronQAAO76471
Porphyromonas gingivalisQAAQ66842
Gloeobacter violaceusQBAC91173
Prochlorococcus marinusQCAE18460
Nostoc sp.QBAB73709
Synechocystis sp.QBAA16871
Fusobacterium nucleatumQAAL94813
Mycoplasma gallisepticumQ
Mycoplasma penetransQBAC43792
Deinococcus radioduransQAAF09595
Bifidobacterium longumQAAN24460
Tropheryma whippleiQAAO44099
Streptomyces coelicolorQCAB92998
Corynebacterium glutamicumQCAF18568
Mycobacterium tuberculosisQAAK44225
Mycobacterium lepraeQCAC29510
Thermoanaerobacter tengcongensisQAAM233
Clostridium perfringensQBAB79708
Clostridium acetobutylicumQAAK77989
Clostridium tetaniQAAO34746
Staphylococcus aureusQBAB93867
Oceanobacillus iheyensisQBAC11958
Bacillus subtilisQCAB11778
Bacillus haloduransQBAB03721
Listeria innocuaQCAC95235
Lactobacillus plantarumQCAD62704
Enterococcus faecalisQAAO79887
Streptococcus agalactiaeQCAD45647
Streptococcus mutansQAAN57795
0.1

Additional Figure 2 (fusA protein)

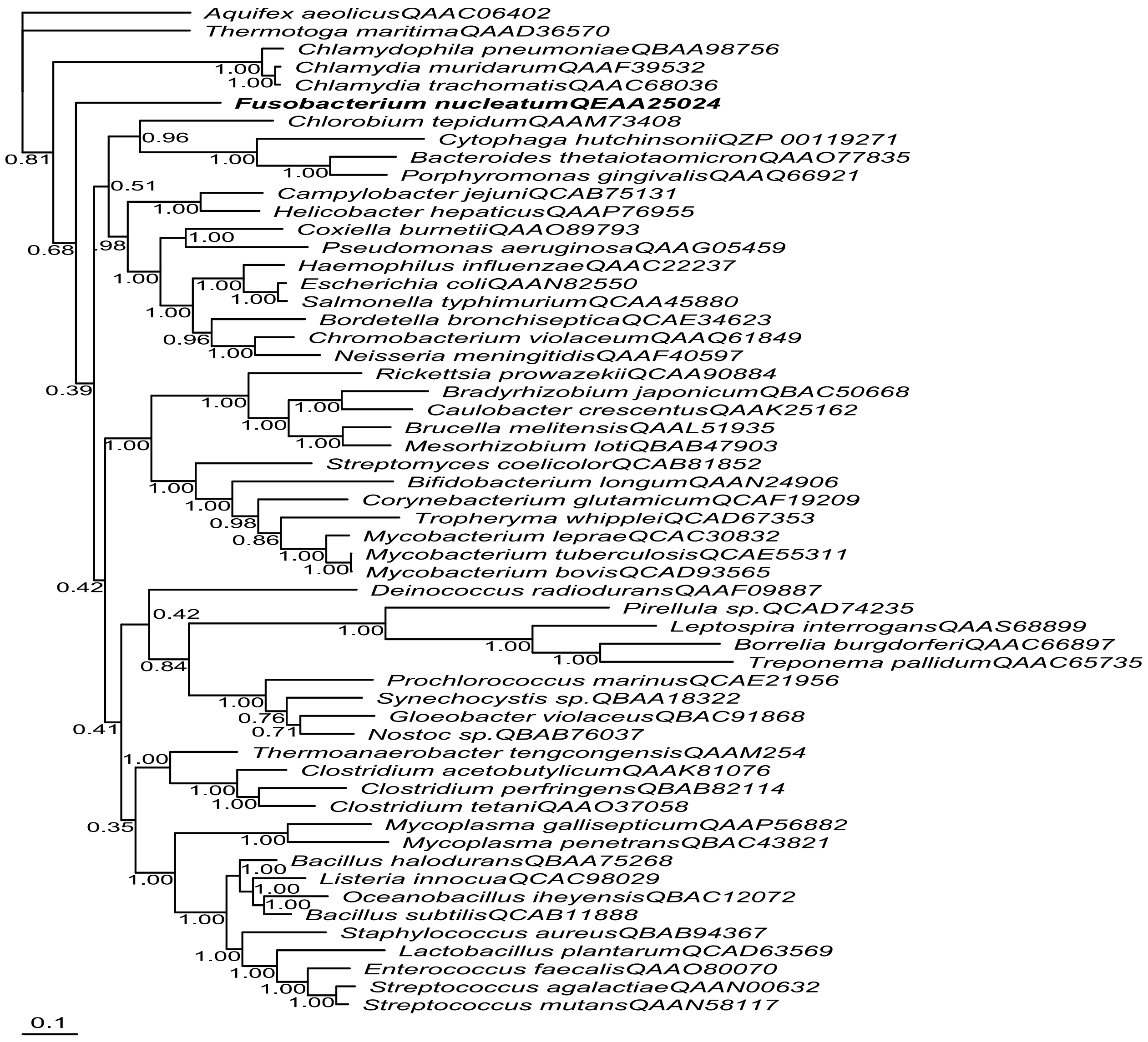

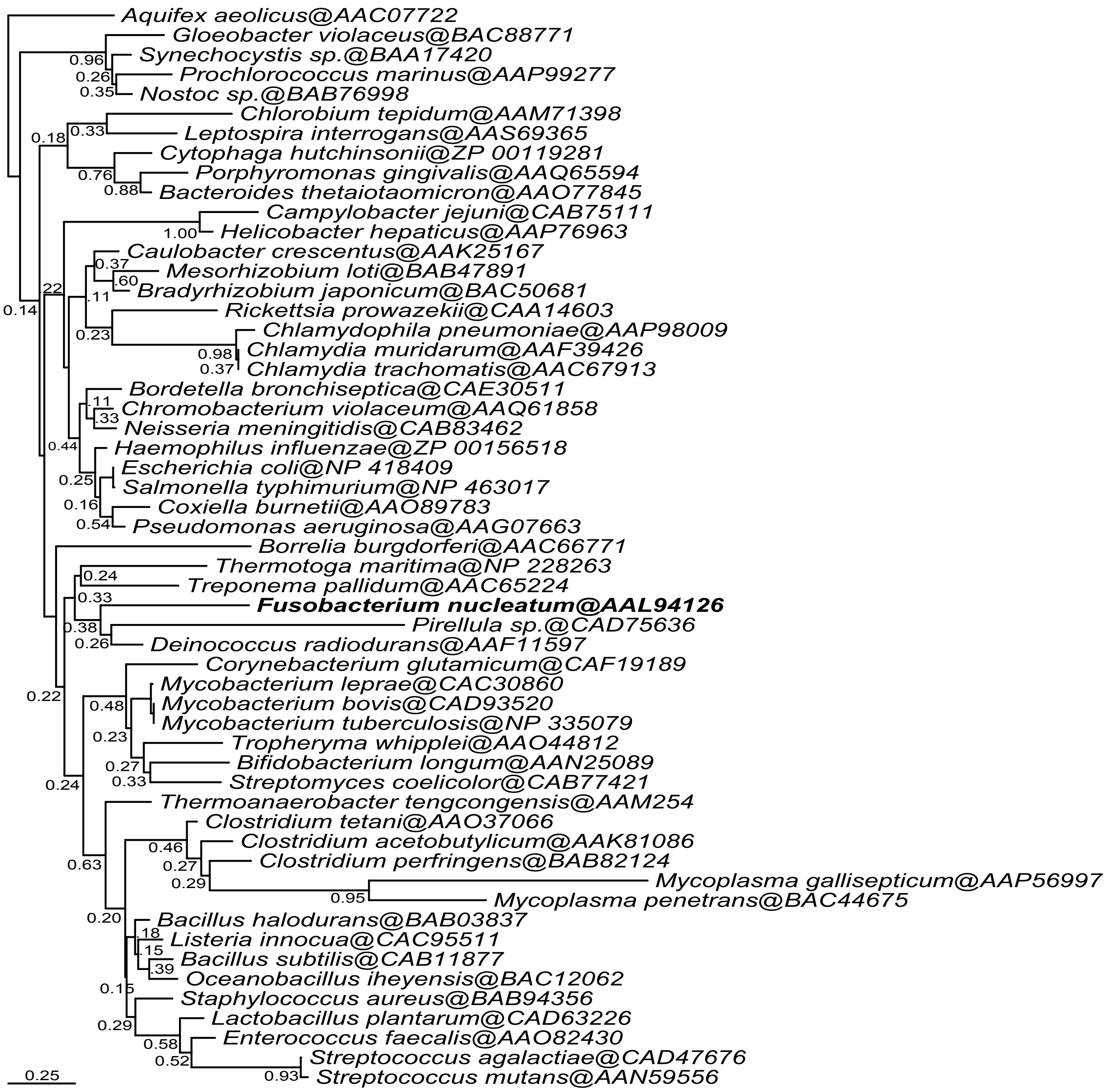
Aquifex aeolicus@AAC07722
Gloeobacter violaceus@BAC88771
Synechocystis sp.@BAA17420
Prochlorococcus marinus@AAP99277
Nostoc sp.@BAB76998
Chlorobium tepidum@AAM71398
Leptospira interrogans@AAS69365
Cytophaga hutchinsonii@ZP 00119281
Porphyromonas gingivalis@AAQ65594
Bacteroides thetaiotaomicron@AAO77845
Campylobacter jejuni@CAB75111
Helicobacter hepaticus@AAP76963
Caulobacter crescentus@AAK25167
Mesorhizobium loti@BAB47891
Bradyrhizobium japonicum@BAC50681
Rickettsia prowazekii@CAA14603
Chlamydophila pneumoniae@AAP98009
Chlamydia muridarum@AAF39426
Chlamydia trachomatis@AAC67913
Bordetella bronchiseptica@CAE30511
Chromobacterium violaceum@AAQ61858
Neisseria meningitidis@CAB83462
Haemophilus influenzae@ZP 00156518
Escherichia coli@NP 418409
Salmonella typhimurium@NP 463017
Coxiella burnetii@AAO89783
Pseudomonas aeruginosa@AAG07663
Borrelia burgdorferi@AAC66771
Thermotoga maritima@NP 228263
Treponema pallidum@AAC65224
Fusobacterium nucleatum@AAL94126
Pirellula sp.@CAD75636
Deinococcus radiodurans@AAF11597
Corynebacterium glutamicum@CAF19189
Mycobacterium leprae@CAC30860
Mycobacterium bovis@CAD93520
Mycobacterium tuberculosis@NP 335079
Tropheryma whipplei@AAO44812
Bifidobacterium longum@AAN25089
Streptomyces coelicolor@CAB77421
Thermoanaerobacter tengcongensis@AAM254
Clostridium tetani@AAO37066
Clostridium acetobutylicum@AAK81086
Clostridium perfringens@BAB82124
Mycoplasma gallisepticum@AAP56997
Mycoplasma penetrans@BAC44675
Bacillus halodurans@BAB03837
Listeria innocua@CAC95511
Bacillus subtilis@CAB11877
Oceanobacillus iheyensis@BAC12062
Staphylococcus aureus@BAB94356
Lactobacillus plantarum@CAD63226
Enterococcus faecalis@AAO82430
Streptococcus agalactiae@CAD47676
Streptococcus mutans@AAN59556
0.96
0.26
0.35
0.33
0.18
0.76
0.88
1.00
0.37
.60
.11
22
0.14
0.23
0.98
0.37
.11
.33
0.44
0.25
0.16
0.54
0.24
0.33
0.38
0.26
0.22
0.48
0.23
0.27
0.33
0.24
0.46
0.27
0.29
0.95
0.63
0.20
.18
.15
.39
0.15
0.29
0.58
0.52
0.93
0.25

Additional Figure 2 (tufA protein)

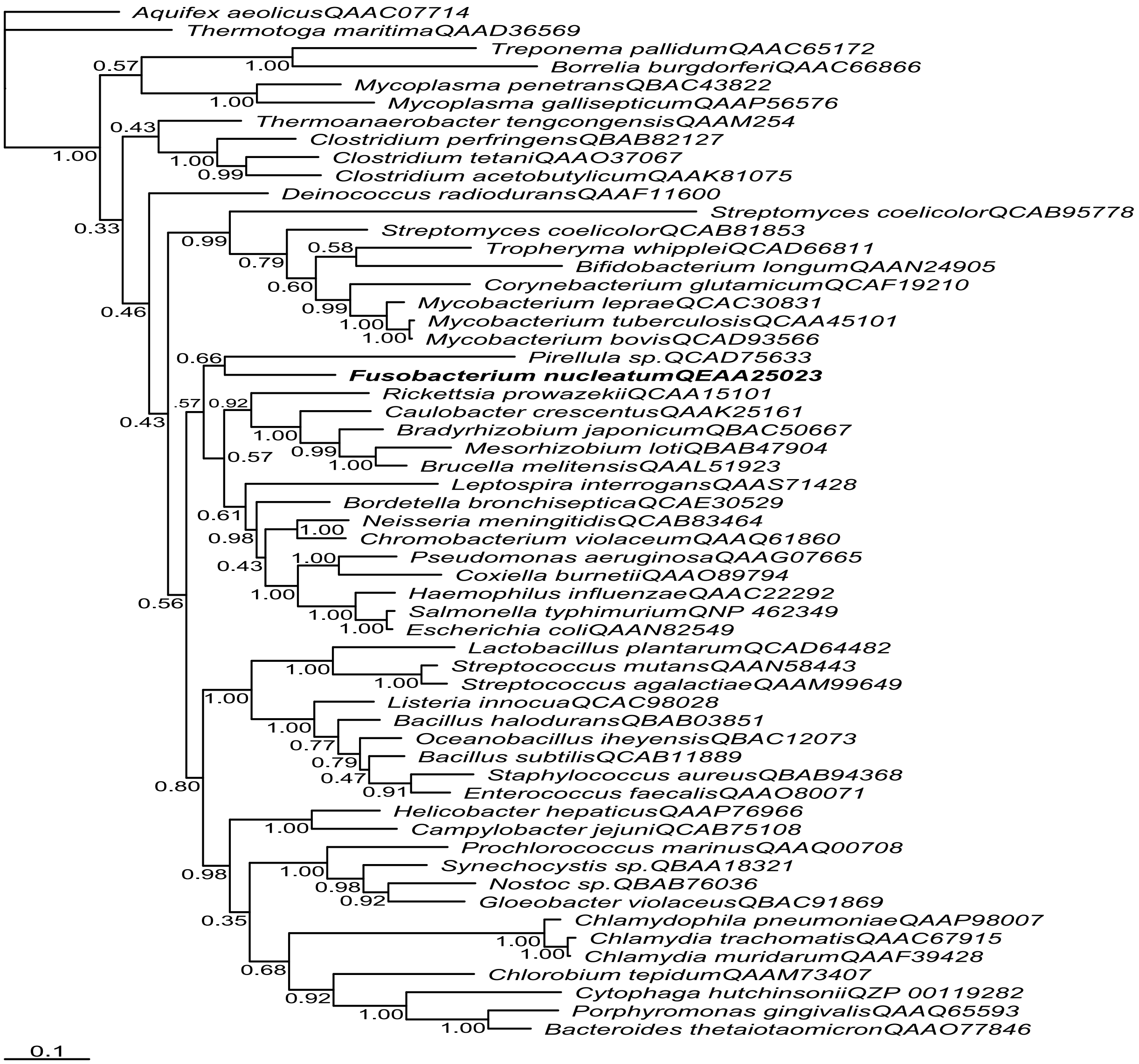

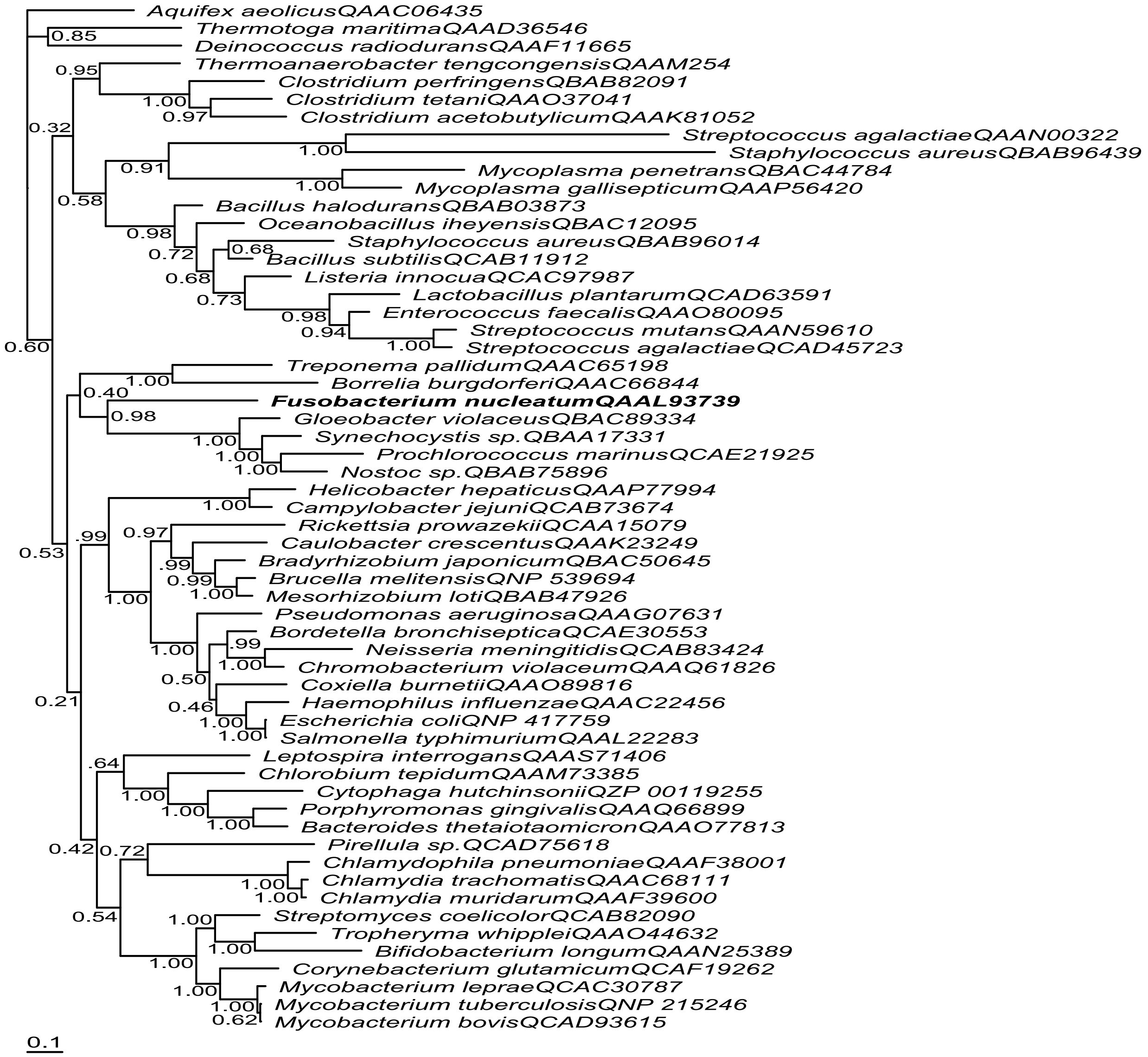
Aquifex aeolicusQAAC06435
Thermotoga maritimaQAAD36546
Deinococcus radioduransQAAF11665
Thermoanaerobacter tengcongensisQAAM254
Clostridium perfringensQBAB82091
Clostridium tetaniQAAO37041
Clostridium acetobutylicumQAAK81052
Streptococcus agalactiaeQAAN00322
Staphylococcus aureusQBAB96439
Mycoplasma penetransQBAC44784
Mycoplasma gallisepticumQAAP56420
Bacillus haloduransQBAB03873
Oceanobacillus iheyensisQBAC12095
Staphylococcus aureusQBAB96014
Bacillus subtilisQCAB11912
Listeria innocuaQCAC97987
Lactobacillus plantarumQCAD63591
Enterococcus faecalisQAAO80095
Streptococcus mutansQAAN59610
Streptococcus agalactiaeQCAD45723
Treponema pallidumQAAC65198
Borrelia burgdorferiQAAC66844
Fusobacterium nucleatumQAAL93739
Gloeobacter violaceusQBAC89334
Synechocystis sp.QBAA17331
Prochlorococcus marinusQCAE21925
Nostoc sp.QBAB75896
Helicobacter hepaticusQAAP77994
Campylobacter jejuniQCAB73674
Rickettsia prowazekiiQCAA15079
Caulobacter crescentusQAAK23249
Bradyrhizobium japonicumQBAC50645
Brucella melitensisQNP 539694
Mesorhizobium lotiQBAB47926
Pseudomonas aeruginosaQAAG07631
Bordetella bronchisepticaQCAE30553
Neisseria meningitidisQCAB83424
Chromobacterium violaceumQAAQ61826
Coxiella burnetiiQAAO89816
Haemophilus influenzaeQAAC22456
Escherichia coliQNP 417759
Salmonella typhimuriumQAAL22283
Leptospira interrogansQAAS71406
Chlorobium tepidumQAAM73385
Cytophaga hutchinsoniiQZP 00119255
Porphyromonas gingivalisQAAQ66899
Bacteroides thetaiotaomicronQAAO77813
Pirellula sp.QCAD75618
Chlamydophila pneumoniaeQAAF38001
Chlamydia trachomatisQAAC68111
Chlamydia muridarumQAAF39600
Streptomyces coelicolorQCAB82090
Tropheryma whippleiQAAO44632
Bifidobacterium longumQAAN25389
Corynebacterium glutamicumQCAF19262
Mycobacterium lepraeQCAC30787
Mycobacterium tuberculosisQNP 215246
Mycobacterium bovisQCAD93615
0.85
0.95
0.32
1.00
0.97
1.00
0.91
1.00
0.58
0.98
0.72
0.68
0.68
0.73
0.98
0.94
1.00
0.60
1.00
0.40
0.98
1.00
1.00
1.00
1.00
.99
0.97
0.53
.99
0.99
1.00
1.00
1.00
.99
1.00
0.50
0.46
1.00
1.00
0.21
.64
1.00
1.00
1.00
0.42
0.72
1.00
1.00
0.54
1.00
1.00
1.00
1.00
1.00
0.62
0.1

Additional Figure 2 (infA protein)

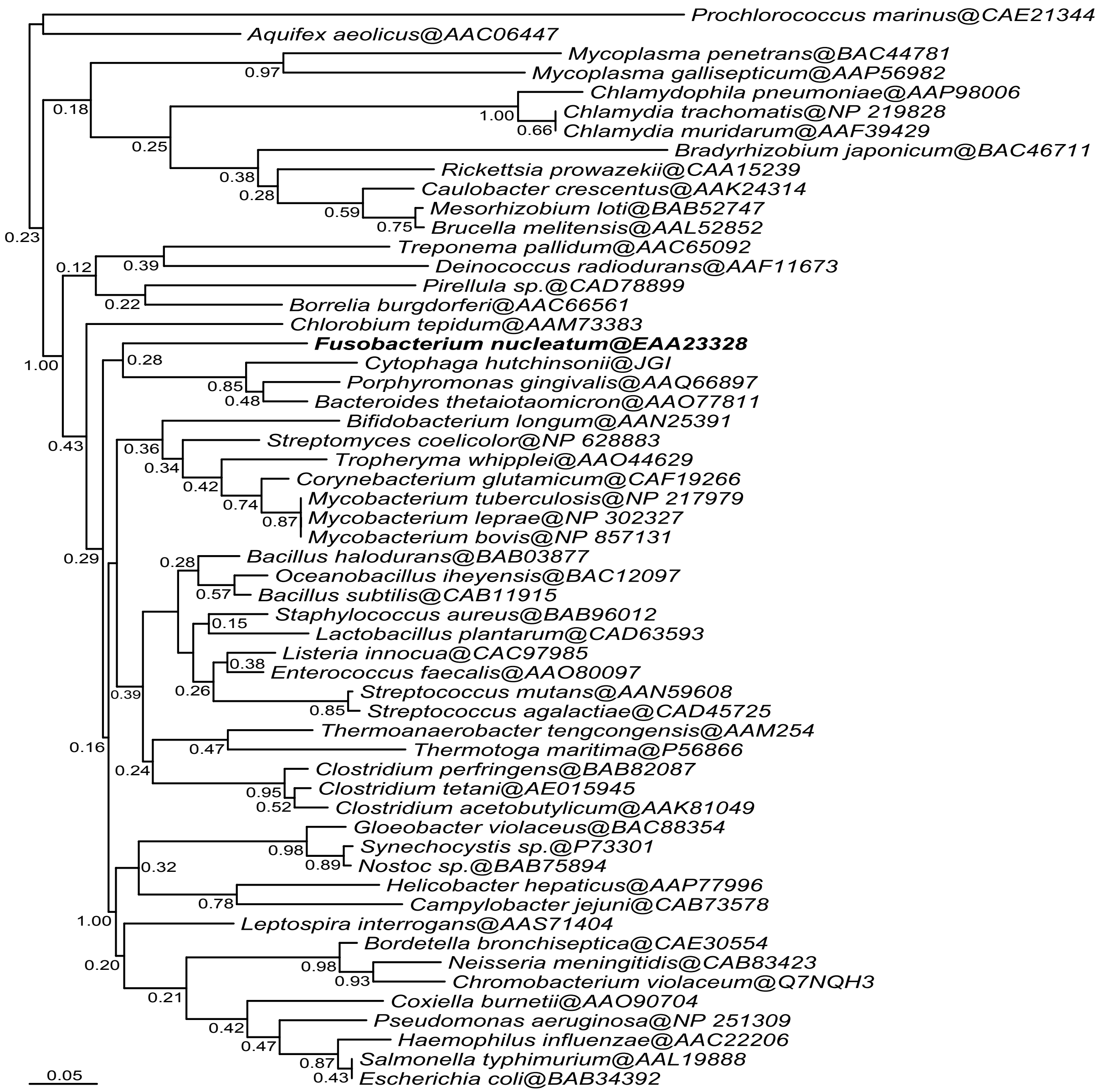

Additional Figure 3

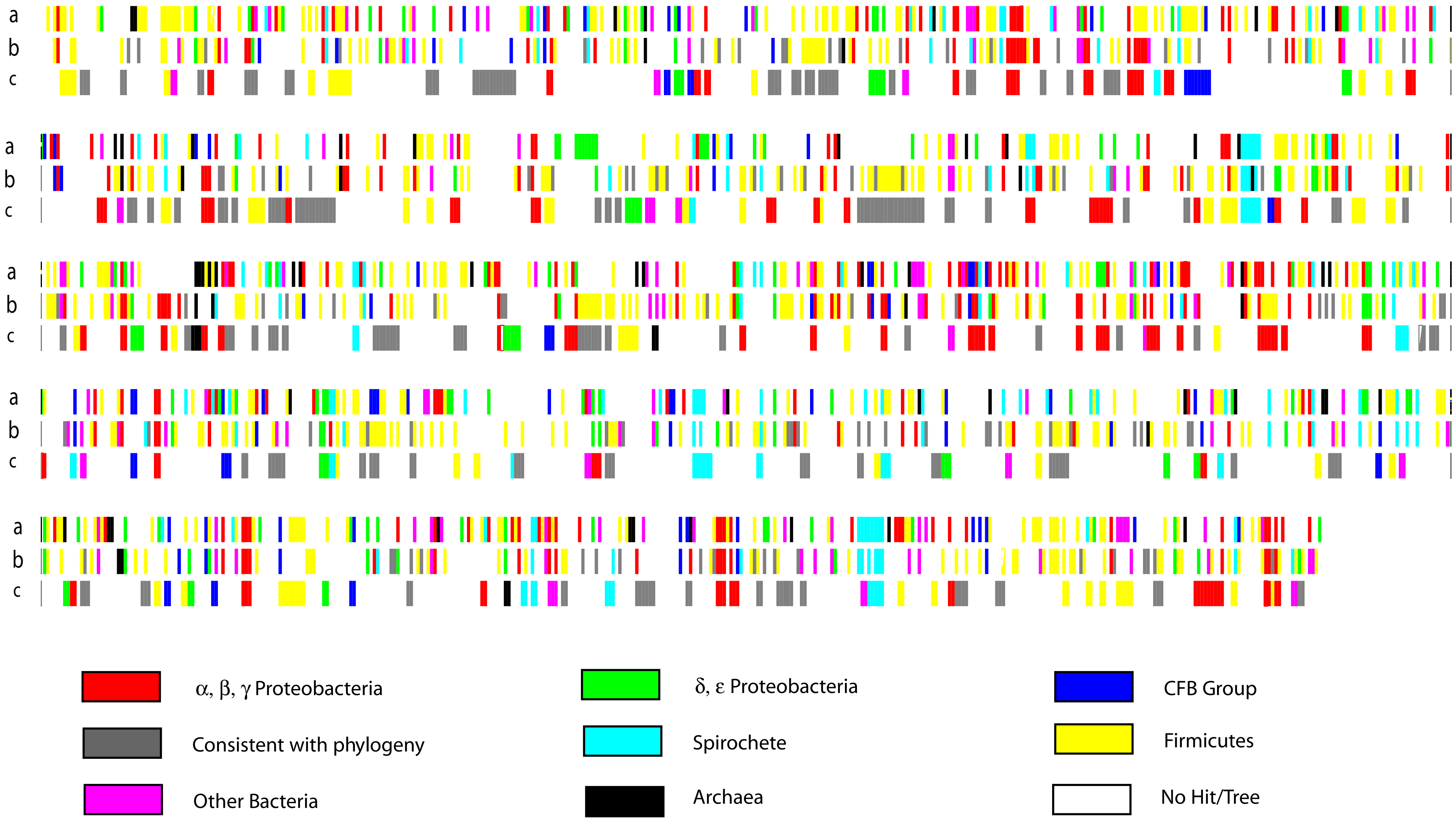

Supplement: Additional File 1 — Supplementary material published as additional information in the manuscript Mira et al. 2004. Evolutionary relationships of Fusobacterium nucleatum based on phylogenetic analysis and comparative genomics. The file contains three additional figures. It is available in pdf format and includes figure legends. [file 1471-2148-4-50-S1.pdf]
